# Evaluation of a prototype array for daily quality assurance in spot scanning proton therapy

**DOI:** 10.1002/acm2.14454

**Published:** 2024-10-02

**Authors:** Veronika Flatten, Henry‐Aravinth Devendranath, Janik Kroh, Matthias Witt, Kilian‐Simon Baumann, Kenneth Gall, Bill Simon, Jörg Wulff, Andreas A. Schoenfeld

**Affiliations:** ^1^ SunNuclear a Mirion Medical Company Melbourne Florida USA; ^2^ Marburg Ion‐Beam Therapy Center (MIT) Marburg Germany; ^3^ Westdeutschen Protonentherapiezentrum Essen (WPE) Essen Germany; ^4^ Heinrich‐Heine University Düsseldorf Dusseldorf Germany; ^5^ Strahlentherapie des MVZ Gesundheit Nordhessen Kassel Germany; ^6^ Institute of Medical Physics and Radiation Protection University of Applied Science Gießen Germany; ^7^ Department for Radiotherapy and Radiooncology Philipps‐University Marburg Marburg Germany

**Keywords:** daily QA, PBS QA, proton therapy

## Abstract

**Background:**

Quality assurance (QA) on a daily basis is an essential task in radiotherapy. In pencil beam scanning proton therapy (PBS), there is a lack of available practical QA devices for routine daily QA in comparison to conventional radiotherapy.

**Purpose:**

The aim was to characterize and evaluate a prototype for the task of daily QA routine for PBS with parameters recommended by the AAPM TG 224, that is, the dose output constancy, the spot position and the distal range verification. Furthermore, a time efficient calibration method for fast and reliable daily QA routine was established for the prototype.

**Methods:**

First, a calibration routine was designed and evaluated, which characterizes the array response and allows for a conversion of the measured signal to clinically needed QA parameters. Finally, a time and resource efficient daily QA routine was developed and tested.

**Results:**

The prototype array can distinguish spot position deviations with sub‐millimeter accuracy, as well as changes in the spot size in terms of FWHM with a 2% sensitivity. The range and thus the energy can be evaluated at different depths also with sub‐millimeter precision. After some training, the setup of the prototype device took roughly two minutes and the total beamtime was about one minute on cyclotron site and five minutes for synchrotrons.

**Conclusions:**

A prototype for daily QA in spot scanning proton therapy was evaluated, which features a fast and easy setup and allows for measuring relevant beam parameters, typically within less than a minute of beam time. All QA parameters as recommended by the AAPM TG 224 report can be analyzed with sufficient accuracy.

## 1 INTRODUCTION

Cancer treatment with protons and ions facilitate the sharp dose fall‐off of the Bragg‐Peak to spare healthy tissue and hence offer a treatment modality to patient with less toxicity in terms of tissue or organ damage and side effects. To take advantage of this high precision treatment, the high accuracy of the beam delivery is essential. Thus, strict quality assurance (QA) protocols need to be in place to ensure a highly conformal dose distribution to the target. In spot scanning proton therapy, the target dose is composed by the cumulation of proton pencil beamlets. To ensure the clinical outcome, each beamlet must be applied with sub‐millimeter precision in terms of its position, its width and its range.

Hence, the QA procedures are typically more time consuming than in conventional radiotherapy. Additionally, dedicated QA devices for proton therapy are recently made available^1^ and also more compact to handle^2^, ^3^, ^4^ but most of the QA is still performed in self designed or diverted photon therapy phantoms rather than with intended QA devices for proton therapy.^5^, ^6^, ^7^, ^8^, ^9^, ^10^, ^11^ In a review study, Ding et al. tackle the problems with QA in proton therapy and identify the limitations of commercially available daily QA systems as a problem for most smaller proton therapy centers.^12^

In addition to this difficulty, there are not as many regulations in place yet as for conventional therapy with photons. Even though QA regulations and recommendation might differ for each country for conventional therapy, regulations specializing on proton beam therapy might not specifically exist. This leaves proton facilities at odds with performing a safe QA, which full‐fills regulatory needs and enables a safe treatment. The most common, internationally used reference guide for QA in proton therapy is the AAPM task group 224 report^13^ “Comprehensive proton therapy machine QA”. It recommends daily, weekly, monthly, and annual QA procedures for all proton beam delivery techniques. Especially for daily QA, the procedure should be fast and concise while not compromising safe operations.

Sun Nuclear, a Mirion Medical Company (Melbourne, FL, USA) has designed a QA device, the Daily QA Proton (DQA‐P), to perform daily quality checks in proton therapy for passive and active scanning facilities. This prototype aims to assist medical physicists, therapists, dosimetrists or technologists to perform a fast and reliable QA routine. This device is aimed to be practical and streamlining QA especially for smaller facilities which do not have resources to develop their own custom equipment.

The AAPM task group 224 report^13^ recommends the dosimetric parameters that need to be checked upon a daily basis: The output constancy, the distal (and proximal) depth verification, and the spot position for pencil beam scanning (PBS). The DQA‐P consists of five clusters of ionization chambers, where each cluster is formed by five ionization chambers covered with different tungsten overlays. By analyzing the signal of these clusters at different water equivalent depths, the parameters of interest (spot size, position, and range) can be compared to a baseline. Hence, the basic parameters can be efficiently checked after a quick setup of the DQA‐P.

In this study, the DQA‐P prototype was evaluated as a daily QA phantom in spot scanning proton therapy. First, the basic relations between the measurement signals and the QA parameters under investigation such as the spot position, the spot size and the beam range were established. Second, a fast and reliable calibration sequence was developed to account for varying beam characteristics of different beamlines. At last, the DQA‐P was tested and analyzed in daily routine.

## 2 MATERIALS AND METHODS

### 2.1 Daily QA‐P prototype

**FIGURE 1:**
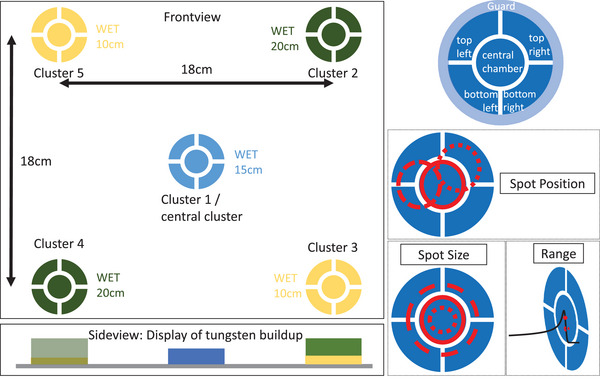
Layout of the DQA‐P: On the left, the array design is presented from a front view and a side view. Different colored clusters indicate a different tungsten build‐up. On the right panel, the idea of this configuration is illustrated: the signal of the surrounding chambers differ depending on the spot size and position. The continuous‐lined red circle indicates the ideal position (top) or extension (middle) or depth with refers to energy (bottom), while the other red circles show exemplary deviations for spot position, spot size and range, respectively. DQA‐P, daily QA proton.

The DQA‐P prototype consists of five fully guarded ionization chamber clusters with a diameter of 3 cm each. Four clusters are positioned each 18 cm apart and the last cluster is centered in the middle of the DQA‐P as shown in Figure 1. Each cluster itself is built of five air filled ionization chambers such that the four outer chambers form a ring surrounding the inner central chamber. The central chamber has a diameter of 1 cm and an active volume of 0.67 ${\mathrm{cm}}^{3}$ while the surrounding chambers have a minimal smaller volume of 0.46 ${\mathrm{cm}}^{3}$. The diameter was chosen to cover the typical range of spot sizes, which means that the surrounding chambers always detect enough signal, even if the spot is shifted. The volume was then a reasonable compromise between the ability to measure the steep dose fall‐off and obtain a sufficient signal.

This design features the ability to simultaneously derive a measure of the spot size and the spot position (see 2.3). Additionally, to allow a range verification combined in a compact design, the chambers are covered with a tungsten layer. While two opposing outer clusters measure at a nominal water equivalent thickness (WET) of 20 cm, the central cluster measures at 15 cm and the other two outer clusters at 10 cm. If the energy of the monoenergetic proton beam is chosen such that the protons stop within the ionization chamber, a minor change of energy, that is, range, is proportional to the measured signal. This means that range can be quickly evaluated through a linear dose to range approximation of the distal part of the Bragg‐peak.

### 2.2 Measurement sites

Measurements and the calibration routine design were performed at the Westdeutsches Protonentherapiezentrum Essen (WPE). The WPE is a cyclotron‐based facility offering actively scanned protons in the clinical range of 100–227 MeV in four rooms.

The measurements were then repeated at the Marburg Ion‐Beam therapy center (MIT) in Germany, which is a synchrotron‐based facility that offers actively scanned proton and carbon ion therapy to patients in three rooms. Proton energies range from 48 to 221 MeV/u and carbon ion energies from 86–430 MeV/u. These measurements confirmed that the device and the developed routines can be transferred to another site. Additionally, it confirmed that the device works with both, a continuous or a pulsed beam.

All measurements were performed at WPE if not mentioned otherwise.

### 2.3 Calibration measurement setup

First, the linearity of the chamber response to the dose rate was verified. Then, a beamtime‐optimized delivery (beam sequence) for a calibration routine was produced. This beam sequence shall allow a fast and reproducible analysis of the correspondence between the detector response and the QA parameter of interest. The QA parameters are described in the following subsections.

**FIGURE 2:**
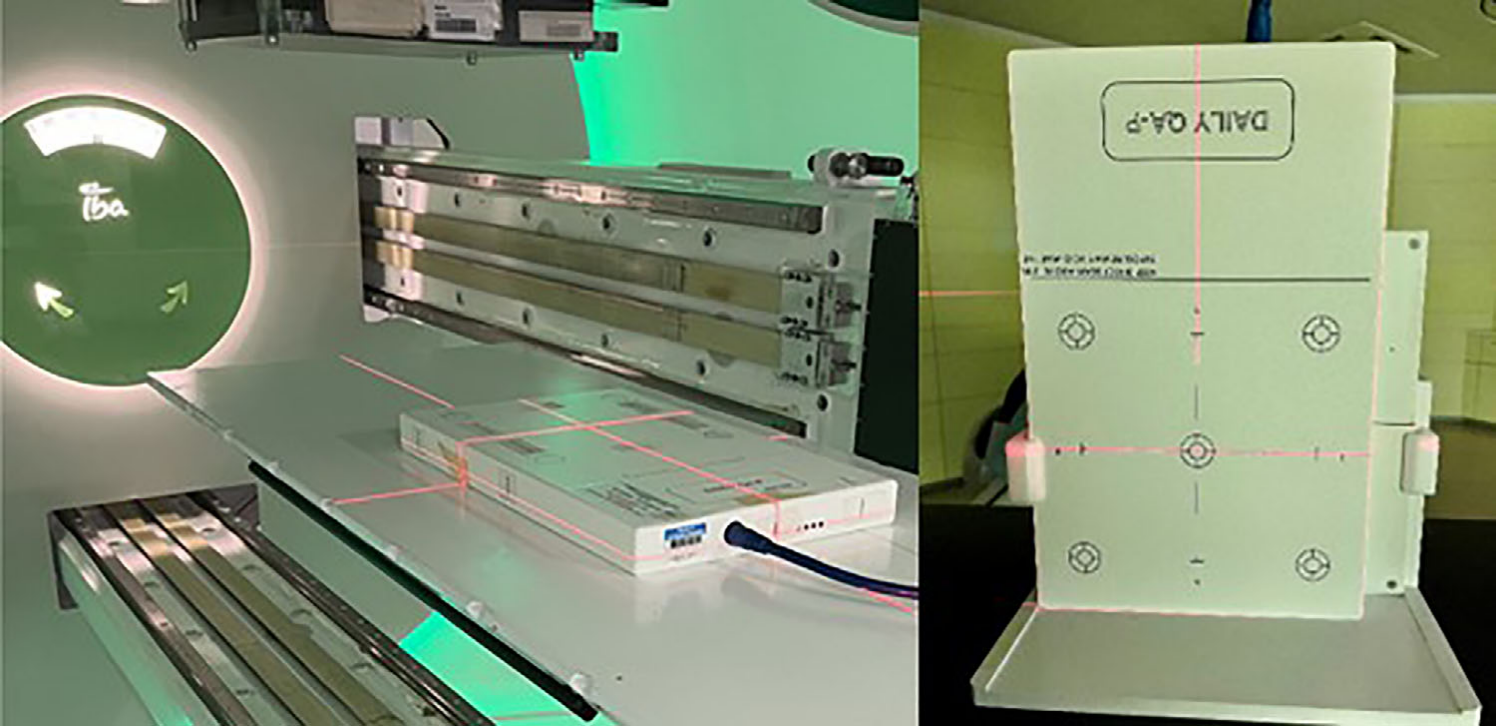
The compact DQA‐P prototype in the measurement position at the proton therapy center in Essen (left) and the center in Marburg (right) in the horizontal beamline setup. DQA‐P, daily QA proton.

In general, the prototype was positioned at isocenter utilizing the room lasers and crosshair markings. The position was checked via additional in‐room imaging via determination of the tungsten slab positions. In a later version, fiducials were added to the prototype design which allow for correct positioning in all three dimensions. For the WPE measurements, the DQA‐P was positioned flat on the treatment couch, while the conformation measurements at MIT were performed with a stand due to the horizontal beam line (see Figure 2).

As this work was performed with a prototype which is still in design, no clinical software accompanied the measurement device. The result analysis and daily trend graph were performed with an external MATLAB routine.

#### 2.3.1 Spot position

In order to determine a change in spot‐position, for each cluster, the different signals of the sides of the outer chambers are used to define two array shift parameters $A_{x}$ and $A_{y}$ as follows:

(1) $$A_{x}=\frac{2\sum_{i\in right}^{2}M_{i}}{\sum_{i\in outer}^{4}M_{i}}$$

(2) $$A_{y}=\frac{2\sum_{i\in top}^{2}M_{i}}{\sum_{i\in outer}^{4}M_{i}}$$

where $M_{i}$ is the signal in a chamber i of one specific cluster, with their position, for example, right in relation to the central chamber (i.e., you can divide the four surrounding (outer) chambers into two right/left or two top/bottom pairs of chambers. The parameter $A_{x}$ and $A_{y}$ change smoothly as a function of position offset. Compared to a baseline value, a value change of $A_{x}$ or $A_{y}$ characterizes a shift in *x* or *y*‐direction, respectively, that is, $\Delta x=f_{x}\left(A_{x}\right)-x_{baseline}$ and $\Delta y=f_{y}\left(A_{y}\right)-y_{baseline}$, where f is the corresponding calibration function.

A radial shift is derived from $A_{x}$ and $A_{y}$ according to

(3) $$\Delta r=\sqrt{\Delta x^{2}+\Delta y^{2}}.$$

The DQA‐P was positioned at the isocenter and each cluster was irradiated with a proton spot of 226.7 MeV, which was the highest available energy at WPE. The maximum energy was chosen to minimize response variance due to potential range deviations. The spot was shifted up to ±4 mm in increments of 1 mm in *x*‐ and *y*‐direction to effectively calibrate over the clinically interesting range.

#### 2.3.2 Spot size

To determine changes in the beam spot size, the array parameter $A_{s}$ is introduced, which correlates the signal of the outer chambers of a cluster to the central cluster:

(4) $$A_{s}=\frac{\sum_{i\in outer}^{4}M_{i}}{M_{central}}$$

where $M_{i}$ are the signals of the four outer chambers and $M_{central}$ is the signal of the central chamber in the same cluster.

To characterize the dependency of the spot size on the parameter $A_{s}$ the array was positioned at different snout‐to‐array distances up to 40 cm from isocenter, employing the beam angular spread to implement different resulting spot sizes. For the final calibration routine, 11 measurement points were chosen and the spot size in air at each point was determined a priori via the treatment planning system calculation and validated with film measurements. As this relation is energy dependent, the calibration was performed for cluster 1 and 5 with a WET of 15 and 10 cm, respectively. This resulted in spot sizes ranging from full‐width at half‐maximum (FWHM) in air of 6.5 up to 10.5 mm and a linear calibration function $f_{s}$ converting $A_{s}$ to a spot size measure even though beam divergence is better described by a linear quadratic fit according to Fermi‐Eyges‐Theory.^14^

#### 2.3.3 Range verification

Each cluster was irradiated with 11 2 cm × 2 cm fields with energies ranging from the maximum energy down to the energy corresponding to a range which stops in front of the chamber, so that the full range of measurable energies is scanned. Table 1 gives the optimized energy selection. Hence, for this prototype version, the minimum energy is roughly 115 MeV corresponding to a range in water of a little bit more that 10 cm.

**TABLE 1:** Energies that were used to scan the full range for each cluster.

	Requested energies/ MeV		
Index	10 cm depth	15 cm depth	20 cm depth
1 ($E_{max}$)	226, 7	226, 7	226, 7
2	134, 7	172, 3	204, 6
3	127, 7	162, 6	192, 9
4	124, 0	157, 5	186, 9
5	120, 3	152, 3	180, 7
6 ($R_{80P}$)	118, 1	149, 0	176, 6
7 ($R_{100}$)	116, 5	147, 0	174, 4
8 ($R_{80D}$)	115, 4	145, 7	172, 9
9	114, 9	145, 0	172, 1
10	114, 4	144, 4	171, 4
11 ($R_{20D}$)	113, 9	143, 8	170, 7

*Note*: The columns depend on the depth of the cluster. The ranges R of interest are given in bracket, where the number gives the percent of maximum dose and D or P denote the distal or proximal region relative to the Bragg Peak.

These data points form a Bragg curve by transforming the energy to range via

(5) $$R=A\cdot E^{b}$$

where A = 0.00244 and b = 1.75 are empirically found parameters.^15^ The Bragg curve can be described analytically by a Bortfeld fit curve.^16^ By implementing a Bortfeld fit, the dose response of the array in dependence of the energy and range is characterized for each beamline and cluster. The obtained Bortfeld fit identifies values like $R_{100}$ or $R_{80D}$ with high precision and by back transformation with Equation (5), the ideal energy for each point in the distal fall‐off can be calculated. For the distal fall‐off, a linear approximation can be fitted. Hence, a change in signal for the specific energy that was determined beforehand can be translated to a change in range, if output correction has been applied.

#### 2.3.4 Dose output

The central cluster was irradiated with 2 cm × 2 cm fields with the maximum energy, which was 226.7 MeV at the WPE cyclotron. Over the range of ± 30% of the nominal dose, a total of 14 dose measurements were conducted: at ± 1%, ± 3%, ±5%, ± 6%,± 10%, ± 18%, and ± 30% of the nominal dose. This check is for consistency only, there is no indication that ion chamber readings should not be linear in dose. In general, dose consistency can be checked with every energy corresponding to a range higher than the WET of the cluster build‐up. For stability and comparability, the highest energy was chosen as this configuration should be most insensitive to changes in range due to the point of measurement in the entrance region and also insensitive to shifts as a field larger than the cluster is used.

### 2.4 Daily dose sequence

After the calibration routine, a daily dose sequence was developed which provides all characterized QA parameters in a short beam sequence. The total dose of the beam sequence was optimized such that the central cluster is irradiated with about 2 Gy physical dose. The daily dose sequence is presented in Table 2: At each cluster, a spot of the highest energy is delivered to check the position and size. This tests the limits of the beam application system as this is the highest proton energy combined with a strong deflection of the scanner magnets to points 9 cm from the central axis.

**TABLE 2:** Overview of the scheme for the daily beam sequence.

QA parameter	Energy	Beam type	Applied to
spot position	maximum energy	spot	center of all clusters
spot size	maximum energy	spot	center of all clusters
spot energy	individually calibrated energy	2 cm field	center of all clusters
dose	maximum energy	2 cm field	central ionization chamber

Abbreviation: QA, quality assurance.

Dose output was validated with a monoenergetic field on the central chamber. The energy was chosen to be the highest available energy.

For the daily analysis of the range, an energy at the turning point of the distal fall‐off, close to the distal dose at 50% of the maximum dose ($R_{50}$) was chosen as reference point as it is very sensitive to changes of beam energy and thus, range. The cluster specific energies corresponding to $R_{50}$ were determined in the calibration process.

To test sensitivity of this constancy check in the daily routine, the beam sequence was delivered and measured for 28 repeated setups on different days. Beam time was less than a minute for the irradiation of the presented beam sequence. To sufficiently test this routine in a case where flags need to be raised by a daily QA device, artificial errors were introduced to the dose sequence, enabling to validate the detection efficiency of the calibrated array. The introduced errors included a variation in the dose output, spot position offsets by deflecting the spots, changes in range by adding 1 mm PMMA (Polymethyl methacrylate) slabs up to 3 mm in front of the device and also a diverted spot size by moving the device downstream the beam axis.

## 3 RESULTS

### 3.1 QA parameters

#### 3.1.1 Spot position

**FIGURE 3:**
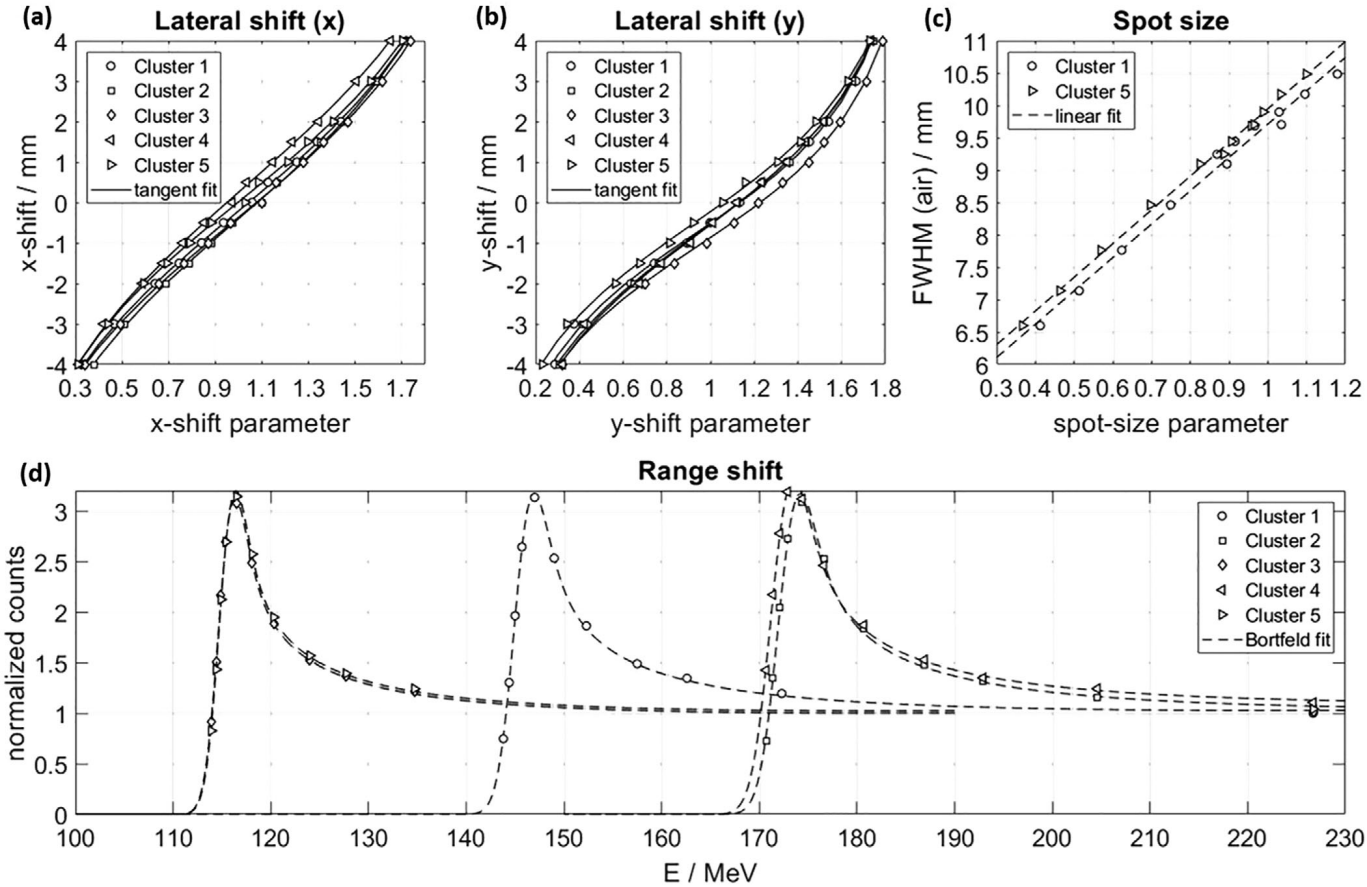
Panel (a) and (b) show the *x*‐ (*y*) shifts of the beam against the array shift parameter $A_{x}$ ($A_{y}$) as defined in Equation (1). A tangent is fitted to all five cluster data sets. Panel (c) presents the base data information of the FWHM in air calculated in the treatment planning system in correlation with the spot size parameter $A_{s}$ for two different clusters with different build‐up depths. $A_{s}$ is defined in Equation (4). A linear fit is plotted. The lower panel (d) shows the obtained Bragg‐Peak for all five clusters by changing the beam energy. All range data are normalized to the highest energy measurement point of Cluster 1. A Bortfeld fit is fitted to all five data sets. Note that the minor offset between the Bragg curves of the lowest build‐up originates from the manufacturing specifications of the height of the tungsten being within 100 μm. FWHM, full‐width at half‐maximum.

The signals of the outer ionization chambers in a cluster change if the spot is not ideally centered on the cluster. Figure 3 panel (a) and (b) show the change in the array parameter $A_{x}$ and $A_{y}$ of each cluster if the spot is shifted in relation to the central position. A tangent fit is presented that allows a conversion from the array shift parameter into a clinical interpretable quantity of a shift in millimeter. The coefficient of determination between the fit and the measurement data was 0.991. For small deviations of the spot position even a linear approximation could be utilized but for higher deviations of the spot position, this would underestimate the shift. By utilizing the tangent, lateral shifts up to 4 mm were analyzed, which is well above the clinically acceptable threshold. The signal correlates such that increments of 0.1 mm shifts are distinguishable.

#### 3.1.2 Spot size

Figure 3 panel (c) shows the array parameter for the spot size $A_{s}$ in dependence on the FWHM in air at the isocenter. The FWHM is well described by a linear function of the measured spot‐size parameter introduced in Equation (4). The coefficient of determination between the fit and the measurement data was 0.983. A 5% change of the spot size would change the array parameter by 10% enabling a sensitive detection of changes in spot size. A higher thickness of build‐up material leads to an slightly increased spot size. However, the slope of the linear approximation is only relevant and as it is consistent, a robust conversion to spot size changes for all clusters is assured. For other centers, a less extensive test with only two points is needed.

No upper limit of spot size was observed, even though the spot is broadened by the tungsten build‐up. As typical spot sizes in FWHM are less than the 3 cm diameter of a cluster, no relevant restrictions ought to appear.

#### 3.1.3 Range verification

Panel (d) in Figure 3 shows the Bragg curves and the fitted Bortfeld fit for all five clusters, where each cluster was irradiated with a sequence of different energies. The steep distal fall‐off results in a high sensitivity to range and thus energy variations.

#### 3.1.4 Dose output

**FIGURE 4:**
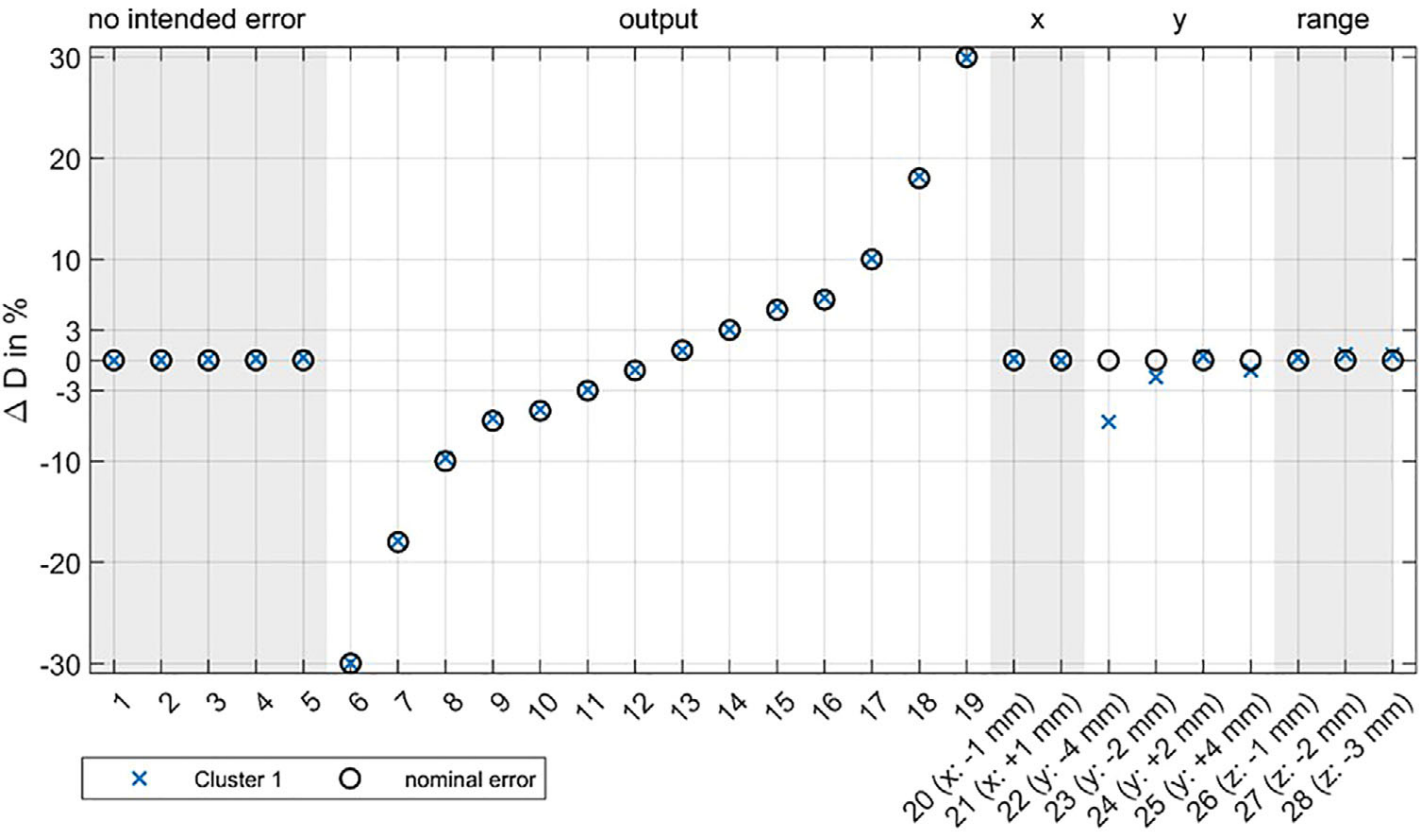
Change of the output (dose) signal over a variety of different constellations. Measurement 1–5 show the output constancy for five different measurement setups (days). 6–19 shows the correlation between the intended change in the dose output (black circles) and the cluster signal. Measurements 20–28 show the effect of a change in other parameters that might influence the dose output measurement.

As the DQA‐P is designed to perform a more efficient daily QA check, the stability over time is crucial as well as the question, if errors would be detected in the routine setup. In Figure 4 the stability of the daily dose output irradiation is shown over 28 measurements, which were performed on 9 days spanning over a few weeks. The first five measurements validate the stability of the dosimetric output. A clear correlation between the dose and the DQA‐P output parameter is observed in measurements 6 to 19. If no other parameters were changed and temperature and pressure correction was applied, the measured output is within 0.5% of the expected value, where the typical fluctuations of the beam output are included.

Only a large beam shift (measurement 22) produced a higher deviation in the dose output value, as the mono‐energetic field no longer covers the whole central chamber used for dose measurement anymore.

The daily dose check can also be performed with spread‐out Bragg Peak(SOBP) fields for field sizes larger than 2 cm. Field sizes are generally limited to a field size of 20 cm to not irradiate the electronic components. However, this agrees with the maximum field size for a lot of centers.

For the prototype analysis, beam interruptions lead to a error in the analysis of the MATLAB analysis routine as this was not a dedicated software which underwent regression testing. For a commercial product, beam interrupts are not expected to cause problems in the analysis.

### 3.2 Design of a calibration routine

The measurement points presented in Figure 3 are put together in three condensed beam sequences. This enables a room specific calibration for the spot shift and energy with only a few beam requests in roughly 6 minutes that needs to be performed once. On the one hand, this increases the accuracy, since spot size and beam divergence may be room specific, and on the other hand, this allows comfortable re‐baselining of the daily QA routine, for example, in case of altered beam characteristics after a repair or maintenance. The array is positioned in isocenter as it would for the daily check and the beam sequence is irradiated. Most of the beam time is needed for the scanned fields of different energies in different clusters as stated in Table 1. The spot size calibration was found to be sufficient as a look‐up table and is not individually determined for each room as inter‐room and inter‐site comparison showed.

This dose sequence was adapted for the synchrotron site and evaluated accordingly. Due to the characteristics of a synchrotron accelerating system, the calibration dose sequence needs longer beam time but in total less than 20 minutes for the full array calibration.

### 3.3 Daily constancy and co‐dependencies

**FIGURE 5:**
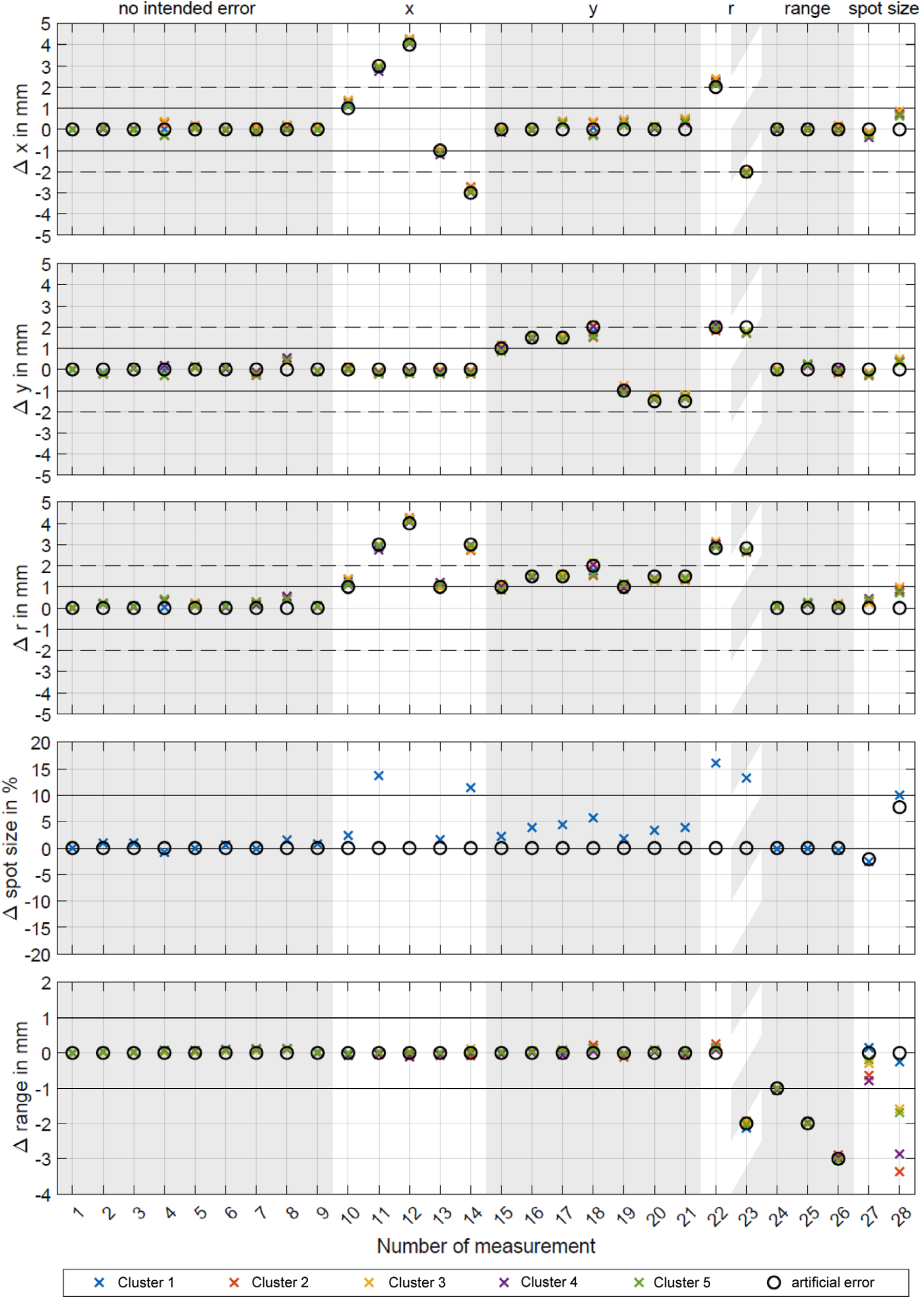
Daily constancy checks over the range of 28 measurement and co‐dependencies. Measurement setups 1–9 show the QA parameters on different days. 10–14 have intended *x*‐shifts of the beam as indicated by the black circles in the top panel and 15–21 intended *y*‐shifts of the beam, respectively. Combined beam shifts in *x* and *y* are visible in the middle panel (Δr) and visible on measurements 22 and 23. Additionally, 23–26 add changes in range (energy) and 27–28 show the effect of a change in spot size. Note that the change in spot size was induced by changing the array to nozzle distance, hence beam divergence affects the range measurement as well as the spot positions on the outer clusters. QA, quality assurance.

Figure 5 shows all parameters that were implemented via the calibration measurements presented in Figure 3 for 28 different irradiations with intentionally introduced delivery errors. The first nine measurements show typical daily variation of the QA parameters without artificially introduced deviations. All data points are within their defined limits and identified correctly as the overlap of the incident value (black circle) and the respective measurement result for each cluster (colored crosses) indicates. Lateral shifts were added in measurements 10 to 23 to analyze the sensitivity and co‐dependencies. The shifts are always identified with a sub‐millimeter precision. Range shifts are identified correctly and with high precision (see measurements 23 to 26). If a spot shift above the threshold is identified, the spot size can not be correctly determined but other parameters such as the range are not influenced. Changes in spot size were also identified within the indicated limits. Since in this study the change in spot size was introduced by repositioning the array downstream of the beam axis outside of the clinically approved region, the additional air affects the range measurement and beam divergence reduces the signal and induces spot shifts (measurements 27 and 28).

### 3.4 Beamline dependencies

**FIGURE 6:**
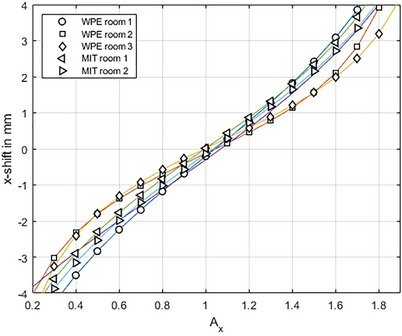
Comparison of the tangent fit function results for different beamlines. Three curves originate from three different beam lines at the WPE cyclotron center and the other two from two different synchrotron beamlines at MIT. WPE, westdeutsches protonentherapiezentrum essen; MIT, Marburg ion‐beam therapy center.

Figure 6 shows the spot shift parameter for five different beamlines, three of which are different rooms of the cyclotron facility WPE and two corresponds to the synchrotron site MIT. There is no visible difference between intra and inter site comparison. It shows that also minor differences in the spot size and shape lead to a different curve shape which can be well represented and accounted for by the calibration routine. This presents the benefits of a room dependent short calibration possibility to increase accuracy.

### 3.5 Activation of the prototype

**FIGURE 7:**
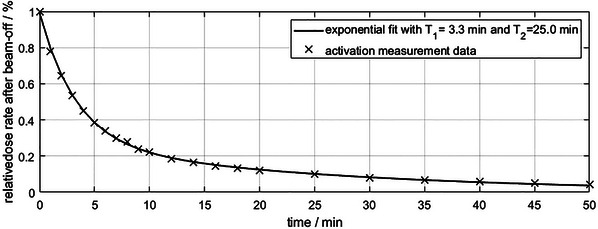
Activation measurement and exponential fit with two decaying components. The half‐life T of the fast‐decaying component is 3.3 minutes and the half‐life of the slower decaying part is 25 minutes.

Since Tungsten is used as build‐up material, the activation dose rate was measured after exposure to 4 Gy, that is, by irradiating the daily QA dose sequence twice. Immediately after irradiation, a dose rate of 42 μSv/h (4.2 mrem/hr) was measured directly at the array's surface above the central cluster. The value reduced to 1.9 μSv/h (0.19 mrem/hr) at the location of the array's electronics, which were not directly exposed. 0.1 μSv/h (0.01 mrem/hr) were measured at the end of the patient table. Some of the activated compounds are fast decaying, exponentially decreasing the dose rate after a few minutes wait as shown in Figure 7. The fit corresponds to a decay of two main components with a small half‐life of 3.3 minutes and a larger half‐life of 25 minutes. After 5 minutes wait, the above mentioned doses are reduced to less than 40%. All measurements that were conducted for this publication showed no additional dose on personal electronic dosimeters or the personal albedo dosimeter badges of the person handling the device.

## 4 DISCUSSION

The implementation of the DQA‐P prototype in the daily clinical routine was found to be fast and efficient. The DQA‐P efficiently measures indicators for QA parameters such as the spot size, the spot position and the range. Minor energy changes show a strong change in the signal for the distal fall‐off enabling a high precision determination of the $R_{50}$ which was chosen as energy dependent QA parameter. Lateral and radial shifts can be identified with sub‐millimeter precision. In theory, a higher accuracy would be achieved by implementing an error‐function which would best describe the (double‐) Gaussian which is scanned over a divided circle. However, in the range of interest, the tangent fit offered an easy, fast, and good approximation as 0.1 mm shifts are distinguishable. The spot size can be determined with uncertainties of about 1% if the spot is centered. However, deviations in the spot size highly increase the uncertainties of all other parameters under investigation. The spot size calculation works as intended for beam shifts of less or equal to 1 mm. The parameters under investigation are in accordance with the TG 224 report for daily QA.^13^ The parameter response differed slightly from beamline to beamline, therefore, the described calibration measurements were performed at four beamlines at two sites increasing the accuracy of the QA parameter conversion for each room. The total setup and delivery time of the calibration routine took less than 10 minutes on a cyclotron site and about 20 minutes on a synchrotron site. The plan used for the daily QA check took less than a minute of beam time.

A dedicated QA device for PBS that is already commercially available is the Sphinx Compact (IBA Dosimetry, Schwarzenbruck, Germany).^2^, ^3^, ^4^ Even though, the concept between the presented prototype and the Sphinx Compact is completely different, the relevant QA parameters recommended by the AAPM report are identified with similar accuracy,^2^ while the array presented in this study features a much smaller form factor and ionization chambers tend to offer a better accumulated dose stability and the ability to directly measure dose.

QA routines for proton therapy are evolving and the dedicated QA devices should and will as well. Staff shortages and the higher cost of proton beam time compared to conventional therapy urges the need for smart, reliable and time‐efficient QA solutions not only for daily QA. Enhancements could be build‐in room devices like the electronic portal imaging device (EPID) which is increasingly used in photon therapy QA as it reduces setup time and uncertainties to a minimum. To test the whole treatment chain; however, devices that are positionable on the treatment couch are needed as well. Also in the future, dedicated software solutions like QA tracking tools and log‐file analysis are on the verge of offering a variety of commercial QA tools for proton therapy as there is for photon therapy.

## 5 CONCLUSION

The integration and performance of prototype for daily QA in spot scanning proton beams was investigated.

Via calibration measurements, a correlation between the array output and the QA parameters under investigation could be established. These measurements also confirmed the sensitivity to effectively determine the QA parameters with a precision well below the daily threshold.

Thereafter, the functionality of the device in the clinical routine was tested with and without the introduction of artificially introduced errors. All introduced delivery errors were correctly identified and the co‐dependencies between the different QA parameters were analyzed.

The DQA‐P was found to be a compact and easy‐to‐use QA device for the daily QA checks full‐filling all required tasks in a simple and efficient way.

## AUTHOR CONTRIBUTIONS

V. Flatten wrote the manuscript draft and performed measurements at the Marburg Ionbeam therapy center together with J. Kroh, M. Witt, and K.‐S. Baumann. They also designed the parameters to be analyzed. H. Devendranath and J. Wulff performed the measurements at the Westdeutsches Protonentherapiezentrum and developed the calibration and daily beam sequence. K. Gall and W. Simon developed the tested prototype and A. A. Schoenfeld coordinated the study design and provided read‐out and data analysis. All authors revised the manuscript, substantively.

## CONFLICT OF INTEREST STATEMENT

The authors declare the following financial interests/personal relationships which may be considered as potential competing interests: V. Flatten, K. Gall, W.Simon, A.A.Schoenfeld report an employment with SunNuclear.

The remaining authors have no conflicts of interest to declare.

## Supporting information

Supporting Information
